# A New Thresholding Method for IR-UWB Radar-Based Detection Applications †

**DOI:** 10.3390/s20082314

**Published:** 2020-04-18

**Authors:** Xuanjun Quan, Jeong Woo Choi, Sung Ho Cho

**Affiliations:** 1 Department of Electronics and Computer Engineering, Hanyang University, 222 Wangsimini-ro, Seongdong-gu, Seoul 04763, Korea; hgkown@hanyang.ac.kr; 2 Xandar Kardian Inc., Seoul 04793, Korea; glen@xandarkardian.com

**Keywords:** UWB, IR-UWB radar, false alarm, miss-detection, threshold, CFAR, detection

## Abstract

In this paper, we proposed a new thresholding method for impulse radio ultra-wideband (IR-UWB) radar-based detection applications by taking both the false alarm and miss-detection rates into consideration. The thresholding algorithm is the key point of the detection application, and there have been numerous studies on these developments. Most of these studies were related to the occurrence of false alarms, such as the constant false alarm rate algorithm (CFAR). However, very few studies have considered miss-detection, which is another crucial issue in detection applications. To mitigate this issue, our proposed algorithm considered miss-detection as well as the false alarms occurring during thresholding. In the proposed algorithm, a threshold is determined by combining a noise signal-based threshold, in which the focus point is the false alarm, with a target signal-based threshold, in which the focus point is a miss-detection, at a designed ratio. Therefore, a threshold can be determined based on the focus point by adjusting the designed ratio. In addition, the proposed algorithm can estimate the false alarm and miss-detection rates for the determined threshold, and thus, the threshold can be objectively set. Moreover, the proposed algorithm is better in terms of understanding the target signal for a given environment. A target signal can be affected by the clutter, installation height, and the angle of the radar, which are factors that noise-oriented algorithms do not consider. As the proposed algorithm analyzed the target signal, these factors were all considered. We analyzed the false alarm and miss-detection rates for the thresholds, which were determined by different combination ratios at various distances, and we experimentally verified the validity of the proposed algorithm.

## 1. Introduction

Due to the increased interest in the Internet of Things (IoT), there has been a growing demand for smart sensors. Smart sensors can be combined with IoT devices, which enables these devices to operate automatically and to provide useful information to the users. In particular, smart sensors are required to implement automation in many areas, such as smart buildings and for security purposes. For example, IoT devices, such as lights and air conditioners, can be combined with smart sensors with presence detection capabilities, allowing them to be turned on or off automatically. Smart sensors with people counting abilities can be used to provide congestion information regarding public places to the users, thereby decreasing congestion and preventing accidents. Smart sensors can also be mounted on vehicles to analyze the surrounding conditions and prevent collisions, and to detect criminal activity, such as intrusions or thefts within security zones.

Thus far, cameras [1,2,3] and infrared sensors [4,5,6] have been mainly used as smart sensors in the IoT market. However, such sensors have certain limitations. Cameras are considered to invade the privacy of others, which has recently become an increasingly important issue, particularly in indoor use. In Europe, the recent implementation of the General Data Protection Regulation (GDPR) [7] has emphasized the importance of privacy. The dependency on light is another limitation of a camera. An infrared sensor is highly sensitive to temperature, and therefore, a new type of smart sensor is needed to overcome these limitations. Radar has recently gained popularity in the IoT market as a smart sensor that can operate without the above limitations.

Radar is a radio frequency (RF)-based sensor that emits electro-magnetic waves and determines the surrounding conditions based on the reflected signals. Radar has commonly been used outdoors, primarily for military purposes, such as in the detection and tracking of planes in the air or ships in the sea. Recently, research on radar is of interest for private use rather than military use, particularly for applications in indoor utilities. For example, radar-based studies on presence detection [8], people counting [9], and positioning [10] are attracting increased attention. Studies have shown the potential of radar in a variety of research fields.

Detection is the most basic and essential technology applied in radar research as most utilities, such as people counting and positioning, operate when the presence of the target is determined. The detection is normally analyzed based on two aspects, false alarm and miss-detection [11]. A false alarm is the indication of the presence of a target when such a target does not exist. In other words, a false alarm indicates a situation in which noise is mistaken to be the target. Thus, the focal point of a false alarm is the noise signal. By contrast, miss-detection is the indication of the absence of a target when the target is present. In other words, a miss-detection indicates the situation in which the target is mistakenly considered to be noise. Thus, the focal point of miss-detection is the target signal.

Most studies on detection have focused on false alarms. The most popular detection algorithm is the constant false alarm rate (CFAR) algorithm [12,13,14], which has been studied ever since the introduction of conventional radar. This algorithm analyzes the statistical characteristics of the noise signal and determines the threshold based on the noise level.

Very few studies have focused on miss-detection. In many cases, miss-detection should be a far more important design factor in determining the threshold for target detection than false alarms. Typically, a false alarm creates an annoying situation requiring a person to confirm whether the triggered situation is genuine. By contrast, a miss-detection may lead to a serious situation, such as collisions between objects or a miss-detection of criminal activity. If we consider the target (mainly humans) and noise signals when determining a threshold, the analysis and prediction results of the cost from a miss-detection or false alarm will be made visible.

The lack consideration of the miss-detection in conventional radar research can be seen in the following aspects. As the conventional radar-based presence detection is mainly used outdoors, there is little clutter other than the target. This results in a relatively high signal-to-noise ratio (SNR). In other words, the strength of the target signal is relatively clear. So, even when considering the noise signal alone, there is a relatively low risk of miss-detection. In addition, it is difficult to reproduce the situation when the target exists in environments such as the sky and the sea. This makes it difficult to collect the target signal that is used in analyzing the miss-detection. Thus, miss-detection has not been previously considered.

This is different from short-range radar, such as impulse radio ultra-wideband (IR-UWB) radar, used in indoors. As the clutter indoors results in a severity of multi-path and low SNR, the strength difference between the target signal and the noise is not as clear as outdoors. In other words, the noise signal is likely to be stronger than the target signal. If a threshold is set by considering only the false alarm, which is determined by the noise signal, there could be a relatively high probability of miss-detection. In addition, it is relatively easy to collect the target signals using short-range indoor radar. It is relatively easy to reproduce the situation when the target exists in an indoor environment; thus, consideration of miss-detection is both necessary and possible for short-range indoor radar.

IR-UWB radar, which uses narrow impulse signals in the time domain and occupies a wide-band frequency in the frequency domain, has been studied in various fields since it was first licensed by the Federal Communications Commission (FCC) for private use in 2002 [15]. As IR-UWB radar occupies a wide-band signal, it generally has to comply with stringent radio regulations. Therefore, IR-UWB radar is mainly used for indoor applications within a distance of 10 m. Owing to its wide bandwidth, this radar has a good penetration rate and high-ranging resolution. It is being studied in various of fields including presence detection [8,16], people counting [9,17], heart rate and respiration rate measurement [18,19,20], radar imaging [21,22], positioning [10,23,24,25], and gesture recognition [26,27].

In Reference [28], an algorithm for extracting valid signals from the received signals of an IR-UWB radar system was considered. However, this algorithm did not distinguish between the absence and presence of a target, but rather distinguished meaningful signals when the target was present. Therefore, in this study, we proposed a new thresholding algorithm for distinguishing between the absence and the presence of a target. In Reference [29], it proposed a miss-detection probability based thresholding algorithm which opens up a new possibility for designing a threshold. We extend Reference [29] to propose a new thresholding method. The proposed algorithm compensates for issues that occur when the CFAR algorithm is used on indoor short-range radar owing to dense clutters. More specifically, the proposed algorithm considers both false alarm and miss-detection rates in determining a threshold by analyzing both the noise and target signals.

This paper is organized as follows. First, the benefits of the work are discussed. Then, we describe the proposed algorithm in detail. Finally, the experiment results are described to verify the validity of the proposed algorithm.

## 2. Discussion on the Benefits of the Work

The performance of detection can be analyzed in two ways. One is a false alarm, which is when the sensor shows a detection when no one is in the detection area. The other is a miss-detection, which occurs when the sensor shows no detection when someone is in the detection area. Most of the studies on detection have been based on the CFAR, which focuses on a false alarm. The CFAR is a noise-oriented thresholding algorithm, which determines a threshold based on the noise signal level. Various types of CFAR algorithms have been proposed, including cell averaging CFAR (CA-CFAR) [30], order static CFAR (OS-CFAR) [31], greatest of CFAR (GO-CFAR) [32], and smallest of CFAR (SO-CFAR) [33].

In a CFAR-based detection algorithm, the threshold is determined by setting a constant false alarm rate. However, the CFAR-based threshold is not set objectively but is set subjectively through numerous experiments. It is difficult to estimate the miss-detection rate for a CFAR-based threshold; thus, the performance in detecting the target must be checked through the experiments. If a high threshold is set, the false alarm rate is decreased while the miss-detection rate is increased. On the other hand, if a low threshold is set, the false alarm rate is increased while the miss-detection rate is decreased. This kind of checking process can be mitigated by considering the target signals as well as the noise signals in determining a threshold, as both the false alarm and miss-detection rates can be estimated for the set threshold. Consideration of both the false alarm and miss-detection rates in determining a threshold allow for threshold adjustments based on the requirements. If the false alarm issue needs to be handled more carefully, the threshold can be adjusted to reduce the false alarm rate. Otherwise, if the miss-detection issue needs to be handled more carefully, the threshold can be adjusted to reduce the miss-detection rate.

The proposed algorithm is also more advantageous to understand the received target signal according to the given environment rather than by simply applying the CFAR algorithm. The proposed algorithm also analyzes the target signal for the given environment that is not considered by the CFAR algorithm. The target signal can be affected by the clutter, the installation height, and the angle of the radar. In the case of clutter, if there is a low amount of clutter, the received signal mainly contains the direct path, which is reflected from the target. On the other hand, if the clutter is dense, the received signal contains not only the direct path reflected from the target but also the multi-path caused by the clutter. For the installation height, the higher the installation height, the farther the distance from the target, and thus the strength of the target signal would be weak, and vice versa. Similarly, if the main lobe of the radar is placed toward the detection area, the strength of the target signal would be strong, and if the side lobe of the radar is placed toward the detection area, the strength of the target signal would be weak. The CFAR algorithm cannot clearly understood this information, as the noise signal shows a similar tendency even in different environment conditions. However, these conditions can be relatively clearly understood by the proposed algorithm as the target signal is taken into consideration.

The proposed algorithm allows for a detectable boundary measurement. The strength of the target signal decreases when the target moves away from the main lobe, which results in a decrease of the detection ability. Therefore, the angle measurement becomes an issue when measuring the detection boundary, especially for the indoor detection case due to the low SNR caused by the clutter. Theoretically, the angle of the antenna is defined as the angle at which the power is attenuated by 3 dB [34], as compared to the main lobe. However, the theoretical angle cannot be used in measuring the detectable boundary. The angle in measuring the detectable boundary is related to the miss-detection rate, because it can be defined as the angle at which the target can be detected at a low miss-detection rate. The CFAR algorithm is based on the noise signal; it cannot address the miss-detection issue, and thus, it cannot be used to measure the detectable boundary for a set threshold. Unlike the CFAR algorithm, the proposed algorithm can handle miss-detection issues, as it takes into account the target signal, which allows the proposed algorithm to measure the detectable boundary for a set threshold.

Although there are many advantages if the miss-detection issue can be considered, thus far, this topic has not been widely studied because it is not necessary for conventional outdoor long range radar. However, for indoor short-range radar, such as IR-UWB radar, miss-detection issues need to be considered.

For an indoor environment, a large number of paths other than the direct path are reflected from stationary clutter. This increases the noise level compared with an outdoor environment, which means the strength difference between the target signal and noise signal becomes smaller. Especially for the case of far-region, the probability that the strength of the noise signal is stronger than the target signal is increased, as the strength of the target signal is attenuated with distance. If a threshold is set by only considering the noise signal, the probability of the miss-detection could be high in the far-region.

In addition, because of the severe multi-paths in an indoor environment, the received signal consists of many clusters when a target is present. This makes the received signal not as clear as the signal in the outdoors, which mainly consists of the direct path. This results in the difficulty of selecting the noise-only signals, which are used in determining a threshold in the CFAR algorithm. In other words, non-noise signals could be included in determining a threshold, which could result in a high threshold. Thus, the probability of miss-detection could increase, because the probability that the target signal is smaller than the threshold increase.

To overcome the defects of the conventional CFAR algorithm in the indoor environment for IR-UWB radar, a moving target CFAR detection algorithm along slow-time profile was proposed in Reference [35]. Slow time is related to the pulse repetition frequency (PRF). The basic idea in Reference [35] was to compare the relative deviation along the slow time with the threshold corresponding to the same fast-time. The fast-time axis is related to the distance. For a moving target, a different fast time is considered at a different slow time, which indicates that the deviation in the slow time is large. Therefore, with the algorithm proposed in Reference [35], the moving target can be detected with a relatively robust performance compared to the conventional CFAR algorithm. However, there remains a lack of consideration regarding miss-detection.

## 3. The Proposed Algorithm

### 3.1. Basic Concept of the Proposed Algorithm

The purpose of the proposed algorithm is to set a threshold by considering both the false alarm and the miss-detection rates rather than only the false alarm rate. The key point of the proposed algorithm is considering not only the noise signal but also the target signal to determine a threshold. To do so, the following steps are required.

1. Data collection—collect the noise and target signals.
2. Probability distribution (PDF) fitting—analyze the statistical distribution of the strength of the collected noise and the target signals.
3. Thresholding through parameter setting—determine noise signal-based threshold by setting the CFAR and target signal-based thresholds through setting the constant miss-detection rate (CMDR). Then final threshold is obtained by combining the determined two thresholds in a designed ratio.

In step 3, setting a target CFAR means setting the target probability of false alarms. The lower the false alarm rate is, the lower the probability of the occurrence of false alarms, and vice versa. Setting a target CMDR means setting the target probability of miss-detection. The lower the miss-detection rate is, the lower the probability of the occurrence of a miss-detection, and vice versa. However, owing to a trade-off relation between the false alarms and miss-detections, a gain of one and a loss of the other occurs.

**Figure 1:**
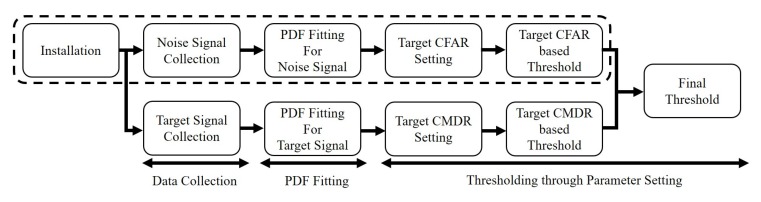
Block diagram of the proposed thresholding algorithm.

Figure 1 shows the block diagram of the proposed algorithm. In the block diagram, the dotted box is the CFAR algorithm. Compared with the CFAR algorithm, the proposed algorithm has one more process that is used to make a threshold based on the target signal, while considering the miss-detection rate. The final threshold is then determined by combining the two thresholds in a designed ratio.

**Figure 2:**
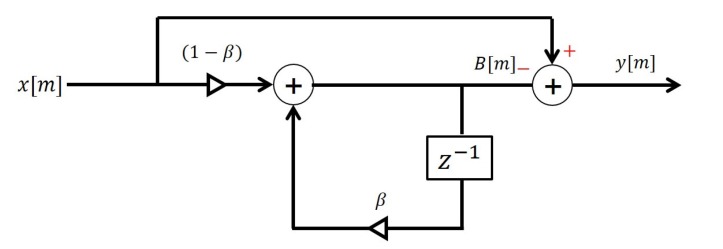
Structure of the running average algorithm.

To verify the validity of the proposed algorithm, we applied the proposed algorithm to an IR-UWB radar. IR-UWB radar operates by transmitting a short-term pulse signal in the time domain and receiving echoes reflected from the environment. The received echoes are then converted into numerous digital samples through an analog-to-digital converter (ADC) with a high sampling rate. The signal, which is converted into a digital signal, can be expressed as follows:

(1) $$\begin{array}{c} x\left[m,n\right]=\sum_{k=1}^{N_{\mathrm{path}}}a_{\mathrm{mk}}s\left[m,n-\tau_{\mathrm{mk}}\right]+N\left[m,n\right], \end{array}$$

where x[m,n] is the digitized received signal, s[m,n] is the transmitted pulse signal, and N[m,n] is the noise. In addition, $a_{\mathrm{mk}}$ and $\tau_{\mathrm{mk}}$ are the scale factor and delay of the received echo, respectively, and *m* and *n* are the slow-time and fast-time indexes. The received signal contains not only the target signal required in the detection but also the unnecessary clutter signal. To extract the target signal, which is the desired signal from the received signal, a clutter removal step is applied. In this study, we used the running average algorithm [36] to remove the clutter signal. The structure of the running average algorithm is shown in Figure 2. We express the clutter removed signal at the *m*-th slow-time as y[m].

(2) $$\begin{array}{c} \begin{array}{cc} B[m] & =\beta B[m-1]+(1-\beta )x[m],\\ y[m] & =x[m]-B[m], \end{array} \end{array}$$

where, B[m] is the *m*-th clutter signal that consists of the previous estimated clutter signal, B[m−1], and the *m*-th received signal x[m]. β is the weighting factor, which is adjustable from zero to one. The lower it is, the greater the weight where the newly received signal is updated to the clutter.

**Figure 3:**
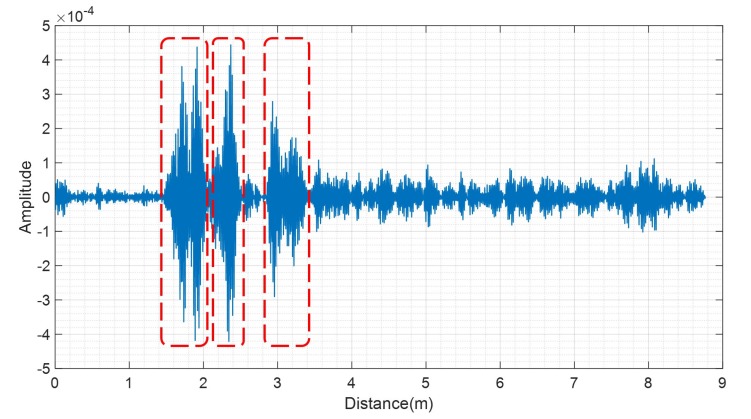
Clutter removed signal when a person is 2 m from the radar.

Figure 3 shows y[m] when a person is 2 m from the radar. In Figure 3, the red boxes represent the received clusters. From Figure 3, we can observe that the received signal reflected from a person contains several clusters. This is because of the multi-path caused by the clutter. In other words, the received signal contains not only the direct path reflected from the person, but also the other path reflected from the clutter.

### 3.2. Detailed Description of the Proposed Algorithm

**Figure 4:**
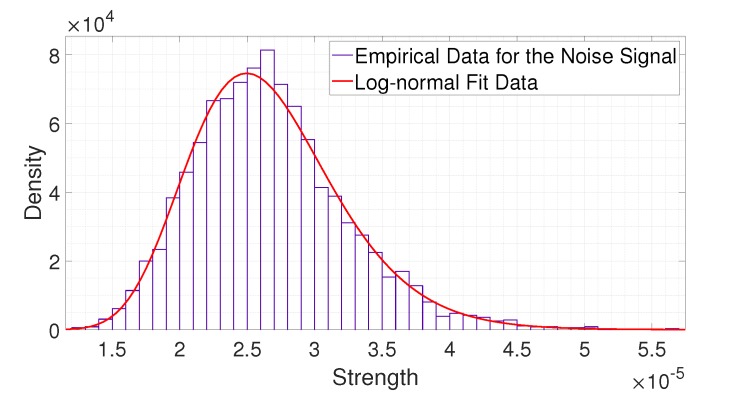
Comparison of the statistical data on the empirical strength with the fitting result for the noise signal.

**Figure 5:**
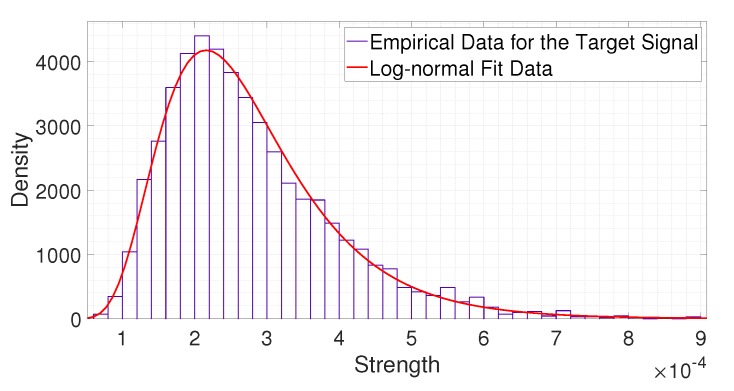
Comparison of the statistical data on the empirical strength with the fitting result for the signal reflected from a person.

1. Data Collection
  To analyze the false alarm and miss-detection rates based thresholds, a data collection of the noise and target signals was required. The noise signals were used to analyze the false alarm rate-based threshold, and the target signals were used to analyze the miss-detection rate-based threshold. The noise signals were collected when there was no one within the detection range of the radar, and the target signals were collected when someone was inside the detection range of the radar. To better understand the characteristics of the target signals, data pertaining to different distances and postures, were collected. The postures can be the direction of the body toward the radar sensor, such as the front of the body, the back of the body, or the sides of the body. The strength of the signal reflected from a person varies with the distance and the posture of the person. The collected noise signal at distance $d_{n}$ can be expressed as follows:
  (3) $$\begin{array}{c} C^{d_{n}}={\left[c_{1,n},c_{2,n},\cdots ,c_{M,n}\right]}^{T}, \end{array}$$
  where *n* is the index under a fast-time, which is related to the distance $d_{n}$. *M* is the number of signals collected along the slow-time index. The collected noise signal C can be expressed as follows:
  (4) $$\begin{array}{c} C=\left\{C^{d_{1}},C^{d_{2}},\cdots ,C^{d_{N}}\right\}, \end{array}$$
  where *N* is the number of samples along the fast-time index.
  The collected signal that is reflected from a person at distance $d_{n}$ can be expressed as follows:
  (5) $$\begin{array}{c} H^{d_{n}}={\left[h_{1,n},h_{2,n},\cdots ,h_{M,n}\right]}^{T}. \end{array}$$
  The collected signal that is reflected from a person can be expressed as follows:
  (6) $$\begin{array}{c} H=\left\{H^{d_{1}},H^{d_{2}},\cdots ,H^{d_{N}}\right\}. \end{array}$$
2. PDF Fitting
  We analyzed the statistical distribution of the collected data. In Reference [28], the authors mentioned that the received signals of IR-UWB radar fit a log-normal distribution. To verify this, we compared the empirical data and log-normal fitting results. Figure 4 and Figure 5 show a comparison of the statistical data with the fitting results. From Figure 4 and Figure 5, we observe that both the noise signal and the signal reflected from a person fit a log-normal distribution. Thus, we apply such a distribution to statistically analyze the received signals.
  The mean and standard deviation of the logarithmic value of a noise signal at distance $d_{n}$, that is, $\mu_{C}^{d_{n}}$ and $\sigma_{C}^{d_{n}}$, can be represented as follows:
  (7) $$\begin{array}{cc} \left[\mu_{C}^{d_{n}},\sigma_{C}^{d_{n}}\right] & =F(C^{d_{n}}),\\ \mu_{C}^{d_{n}} & =\mathrm{mean}(log\left(C^{d_{n}}\right)),\\ \sigma_{C}^{d_{n}} & =\sqrt{\mathrm{var}(log\left(C^{d_{n}}\right))}. \end{array}$$
  The mean and standard deviation of the logarithmic value of the signal reflected from a person at distance $d_{n}$, that is, $\mu_{H}^{d_{n}}$ and $\sigma_{H}^{d_{n}}$, can be represented as follows:
  (8) $$\begin{array}{cc} \left[\mu_{H}^{d_{n}},\sigma_{H}^{d_{n}}\right] & =F(H^{d_{n}}),\\ \mu_{H}^{d_{n}} & =\mathrm{mean}(log\left(H^{d_{n}}\right)),\\ \sigma_{H}^{d_{n}} & =\sqrt{\mathrm{var}(log\left(H^{d_{n}}\right))}. \end{array}$$
3. Thresholding through Parameter Setting
  As described before, we obtained the statistical distribution of the noise signals and the signal reflected from a person for the given environment. Based on this information, a threshold was determined by setting a target CFAR, $P_{Fa}$ and target CMDR, $P_{Md}$. Once $P_{Fa}$ and $P_{Md}$ were set, we obtained two types of threshold, a $P_{Fa}$-based threshold, which was focused on the noise signal, and a $P_{Md}$-based threshold, which was focused on the signal reflected from a person. Unlike the CFAR algorithm, the algorithm proposed in this study considers both conditions in thresholding because if only one condition is considered, the threshold is only optimized for that condition and the other is sacrificed. The threshold $T_{Fa}$, which is based on $P_{Fa}$, can be expressed through the following equation:
  (9) $$\begin{array}{cc} P_{Fa}= & 1-\int_{0}^{T_{Fa}}\frac{1}{\sqrt{2\pi }x\sigma_{C}}e^{-\frac{{}^{\left(\ln^{x}-\mu_{C}\right){}^{2}}}{2{\left(\sigma_{C}\right)}^{2}}}dx\\ = & \frac{1}{2}-\frac{1}{2}\mathrm{erf}\left(\frac{\ln^{T_{Fa}\left(P_{Fa}\right)}-\mu_{C}}{\sqrt{2}\sigma_{C}}\right), \end{array}$$
  (10) $$\begin{array}{cc} T_{Fa}= & F\left(P_{Fa},\mu_{C},\sigma_{C}\right)\\ = & e^{\sqrt{2}\sigma_{C}{\mathrm{erf}}^{-1}\left(1-2P_{Fa}\right)+\mu_{C}}, \end{array}$$
  where erf and ${erf}^{-1}$ are the error function and inverse error function, respectively.
  The threshold $T_{Md}$, which is based on $P_{Md}$, can be expressed in following equation:
  (11) $$\begin{array}{cc} P_{Md}= & \int_{0}^{T_{Md}}\frac{1}{\sqrt{2\pi }x\sigma_{H}}e^{-\frac{{}^{\left(\ln^{x}-\mu_{H}\right){}^{2}}}{2{\left(\sigma_{H}\right)}^{2}}}dx\\ = & \frac{1}{2}+\frac{1}{2}\mathrm{erf}\left(\frac{\ln^{T_{Md}\left(P_{Md}\right)}-\mu_{H}}{\sqrt{2}\sigma_{H}}\right), \end{array}$$
  (12) $$\begin{array}{cc} T_{Md}= & F\left(P_{Md},\mu_{H},\sigma_{H}\right)\\ = & e^{\sqrt{2}\sigma_{H}{\mathrm{erf}}^{-1}\left(2P_{Md}-1\right)+\mu_{H}}. \end{array}$$
  The final threshold $T_{F}$, which is based on $T_{Fa}$ and $T_{Md}$, can be expressed as follows:
  (13) $$\begin{array}{cc} T_{F}= & F\left(\alpha ,T_{Fa},T_{Md}\right)\\ = & \alpha \;T_{Fa}+\left(1-\alpha \right)\;T_{Md}\\ = & \alpha \;e^{\sqrt{2}\sigma_{C}{\mathrm{erf}}^{-1}\left(1-2P_{Fa}\right)+\mu_{C}}\\ \; & +\left(1-\alpha \right)\;e^{\sqrt{2}\sigma_{H}{\mathrm{erf}}^{-1}\left(2P_{Md}-1\right)+\mu_{H}}, \end{array}$$
  where α is the weight factor used to determine the final threshold, which is adjustable from zero to one. For α equal to zero, $T_{F}$ is the same as $T_{Md}$, which means that the threshold is determined based only on the miss-detection rate. For α equal to one, $T_{F}$ is the same as $T_{Fa}$, which means the threshold is determined based only on the false alarm rate, and thus it is same with the CFAR algorithm. The smaller α is, the greater the weight of the miss-detection rate, and vice versa. If the false alarm is an issue that should be handled more carefully, α can be set to a large value; otherwise, α can be set to a small value. Therefore, with the proposed algorithm, it is possible to set a threshold according the focusing point.

**Figure 6:**
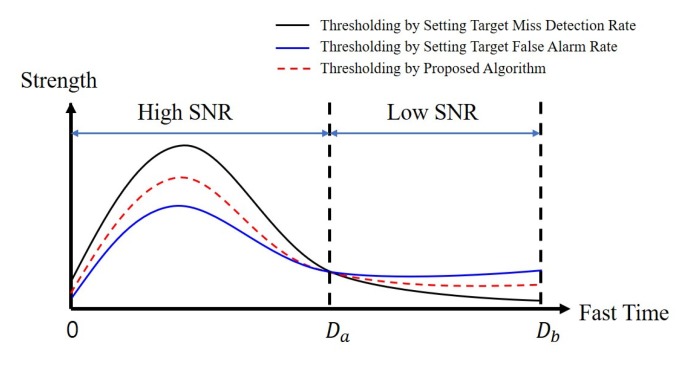
Example of the proposed thresholding algorithm.

Figure 6 shows an example of the proposed algorithm. In Figure 6, the black line refers to $T_{Md}$, the blue line refers to $T_{Fa}$, and the red dotted line refers to $T_{F}$. As shown in the figure, $T_{Md}$ is greater than the $T_{Fa}$ from zero to $D_{a}$, and $T_{Md}$ is smaller than the $T_{Fa}$ from $D_{a}$ to $D_{b}$. Herein, we call the distance from zero to $D_{a}$ as the near-region, and the distance from $D_{a}$ to $D_{b}$ as the far-region. The SNR is high in the near-region, and the signal reflected from a person is stronger than the noise signal, which results in $T_{Fa}$ being lower than $T_{Md}$.

This means that a threshold can be designed to $T_{Fa}$, as $T_{Fa}$ has satisfied the target miss-detection rate. We can see that there is a margin between $T_{Fa}$ and $T_{Md}$ at the near-region, which means that a new threshold can be designed to further reduce false alarm and miss-detection rates when compared to target values. However, this cannot be done with $T_{Fa}$ alone, because we do not know how much the threshold can be adjusted without increasing the miss-detection rate compared with the target value. If we can know the margin between the strength of the signal reflected from a person and the strength of the noise signal, this work becomes possible, since the threshold can be adjusted between $T_{Fa}$ and $T_{Md}$.

For the far-region, the signal is weaker than that of the near-region, and thus the threshold is smaller in the far-region. In addition, owing to the low SNR in the far-region, the probability that the noise signal is stronger than the signal reflected from the person is relatively high compared to the near-region. To maintain the target false alarm and miss-detection rates, $T_{Fa}$ could be higher than $T_{Md}$ in the far-region. Thus, it becomes not possible to satisfy both the target false alarm and the target miss-detection rates as with the near-region. If $T_{Fa}$ is used as the final threshold, the false alarm rate can meet the target value, but the miss-detection rate would be higher than the target value. If $T_{Md}$ is used as the final threshold, the miss-detection rate can meet the target value, but the false alarm rate would be higher than the target value. However, it is possible to adjust the threshold accordingly for the purpose.

If the focus should be placed on the false alarm, the threshold can be adjusted to reduce the false alarm rate by setting the weight factor to a large value, then the miss-detection rate for the set threshold can be estimated. If focus should be placed on the miss-detection, the threshold can be adjusted to reduce the miss-detection rate by setting the weight factor to a small value, then the false alarm rate for the set threshold can be estimated. This means a threshold can be determined by comparing the estimated false alarm and miss-detection rates. Therefore, a context-sensitive threshold can be set.

The detailed logic flow of the proposed algorithm is summarized in Algorithm 1.
**Algorithm 1** Proposed Algorithm1:**procedure** (y[m])2:    Data Collection▹ data collection from y[m];3:    C←collectednoisesignal;4:    H←collectedsignalwhichisreflectedfromhuman;5: 6:    N←digitalsamplesalongfasttime7:    settargetfalsealarmrate$\left(P_{Fa}\right)$8:    settargetmiss-detectionrate$\left(P_{Md}\right)$9:    setweightfactor(α)10:    **for** n←1 to *N* **do**11:        PDF Fitting12:        $\left[\mu_{C}^{d_{n}},\sigma_{C}^{d_{n}}]\leftarrow F(C^{d_{n}}\right)$13:        $\left[\mu_{H}^{d_{n}},\sigma_{H}^{d_{n}}]\leftarrow F(H^{d_{n}}\right)$14: 15:        Thresholding Via Parameter Setting16:        $T_{Fa}^{d_{n}}=F(P_{Fa},\mu_{C}^{d_{n}},\sigma_{C}^{d_{n}})$17:        $T_{Md}^{d_{n}}=F(P_{Md},\mu_{H}^{d_{n}},\sigma_{H}^{d_{n}})$18:        $T_{F}^{d_{n}}=\alpha \times T_{Fa}^{d_{n}}+\left(1-\alpha \right)\times T_{Md}^{d_{n}}$    19:    **return** $T_{F}^{d_{1}},T_{F}^{d_{2}},\ldots ,T_{F}^{d_{N}}$

## 4. Experiment Results

### 4.1. Experiment Configuration and Validation

To show the validity of the proposed algorithm, we conducted experiments in different environments with different clutter level and different number of people, using an IR-UWB radar with a Xethru X4 chip developed by NOVELDA in Norway. The center frequency and −10 dB bandwidth were 8.748 and 1.5 GHz, respectively [37]. During the experiments, the frame rate was approximately 20 frames per second (fps).

#### 4.1.1. Experiment in a Light Clutter Environment

**Figure 7:**
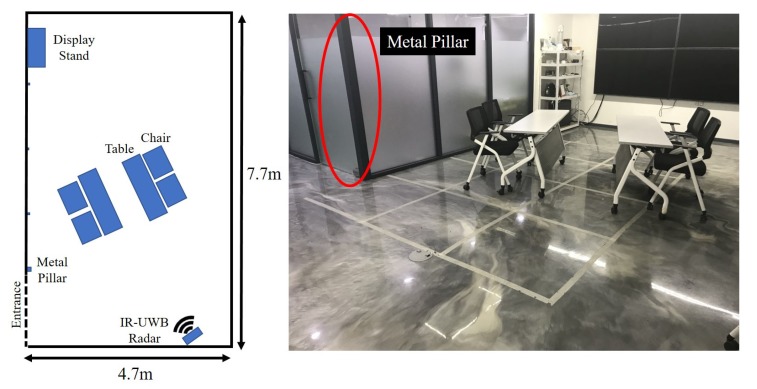
Light clutter environment.

Figure 7 shows the experiment environment with light clutter. A normal meeting room environment was used with a metal pillar at a position of 4 m away. To analyze both the false alarm and the miss-detection rate-based thresholds, we gathered two types of data for a certain time period in that environment, one for noise and the other for a person at different distances with various poses. The gathered data were then used to analyze the statistical distribution.

**Figure 8:**
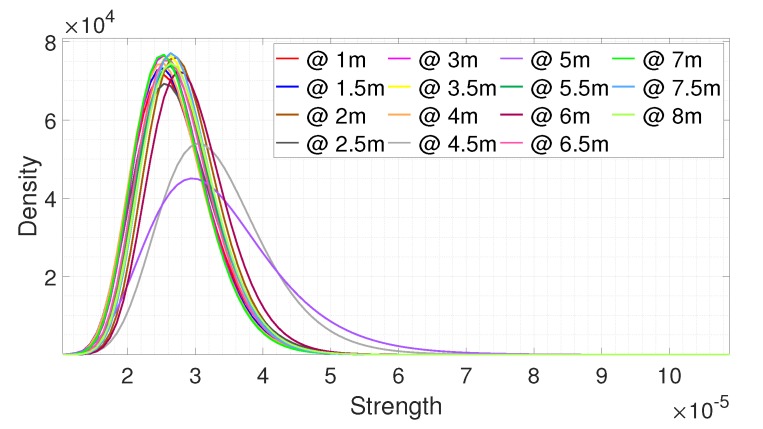
The fitting results for the noise signals at different distances.

**Figure 9:**
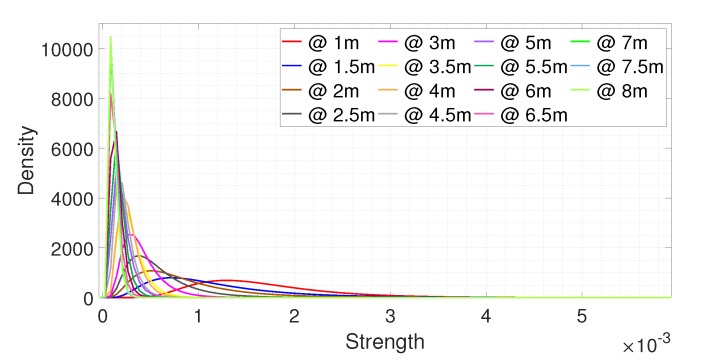
The fitting results for the signals reflected from the person at different distances.

Figure 8 and Figure 9 show the fitting results of the data collected in the experiment environment according to the distance. Figure 8 shows the fitting results for the noise signals collected from 1 m in 0.5 m increments. We observed that the statistical distribution of the noise signal was similar even at different distances. The fitting results at 4 m and 5 m were biased to the right as compared to the other results, which means that the signal strengths at 4 m and 5 m were stronger than those of the other distances. The reflected signal was strong as there was a metal pillar located at that position. Figure 9 shows the fitting results for the signals reflected from a person at different distances. The data was collected from 1 m to 8 m in 0.5 m increments with different postures. In Figure 9, the fitting results are shifted to the left as the distance increased, which means the strength of the signal reflected from a person decreased as the distance increased. It can be seen that with the noise signal there was a limitation on understanding the received target signal, as the trend of the target signal cannot be predicted.

**Figure 10:**
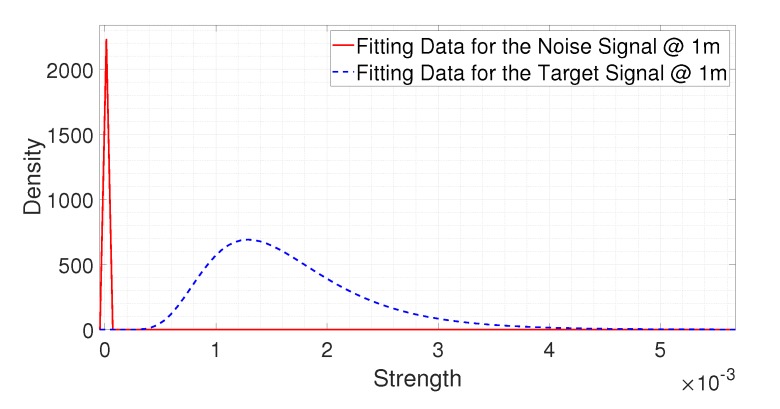
The fitting results for the noise signal and the signal reflected from the person at 1 m.

**Figure 11:**
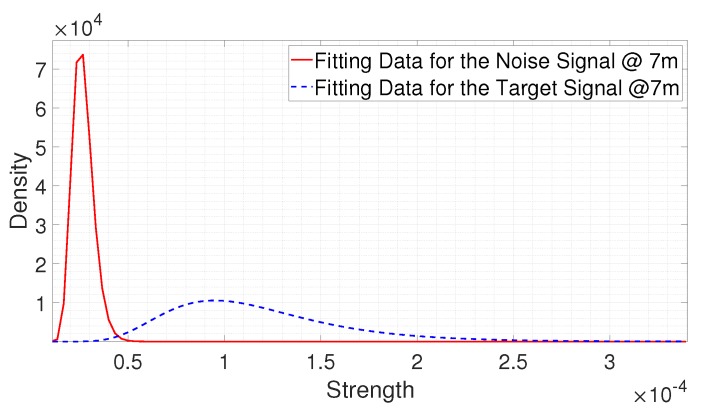
The fitting results for the noise signal and the signal reflected from the person at 7 m.

Figure 10 and Figure 11 show the fitting results for the noise signal and the signal reflected from a person at different distances. Figure 10 shows the fitting results at 1 m. In Figure 10, the red line indicates the fitting result of the noise signal, and the blue dotted line shows the fitting result of the signal reflected from a person. We can observe that there is a large gap between the two fitting results. This indicates that the strength of the signal reflected from a person was much stronger than the strength of the noise signal at 1 m, which means the SNR was high at this certain distance. In this case, the probability that $T_{Md}$ is larger than $T_{Fa}$ is high. So even if the threshold was determined by only considering the false alarm rate, both the target false alarm and the miss detection rates could be satisfied. However, if the strength difference between the two kinds of threshold can be known, then it is possible to design a new threshold, which is greater than $T_{Fa}$ and smaller than $T_{Md}$, to further reduce the false alarm and miss detection rates compared to the target values.

Figure 11 shows the fitting results at 7 m. As shown in Figure 11, there are overlapping parts between the two fitting results, which means that the probability that the signal reflected from a person is weaker than the noise signal is relatively high. We can state that the SNR is low at this certain distance. The larger the overlapping part, the lower the SNR. In this case, in order to satisfy the target false alarm and miss-detection rates, $T_{Fa}$ could be set to a large value and $T_{Md}$ could be set to a small value. However, because of the low SNR, it is more likely that $T_{Fa}$ is greater than $T_{Md}$, making it difficult to satisfy both the target false alarm and miss detection rates simultaneously. If a threshold is set to satisfy the target false alarm rate, the sacrifice of the miss-detection rate is inevitable. As a threshold is set to reduce the false alarm rate, the threshold can be set to relatively high, which could result in the increase of the miss-detection rate compared to the target value. If a threshold is set to satisfy the target miss-detection rate, the sacrifice of the false alarm rate is inevitable. As a threshold is set to reduce the miss-detection rate, the threshold could be set to relatively low, which could result in the increase of the false alarm rate compared to the target value. Therefore, a compromise between the false alarm rate and the miss-detection rate is needed based on the requirements. This can be achieved with the proposed algorithm by comparing the estimated false alarm and miss-detection rates.

**Figure 12:**
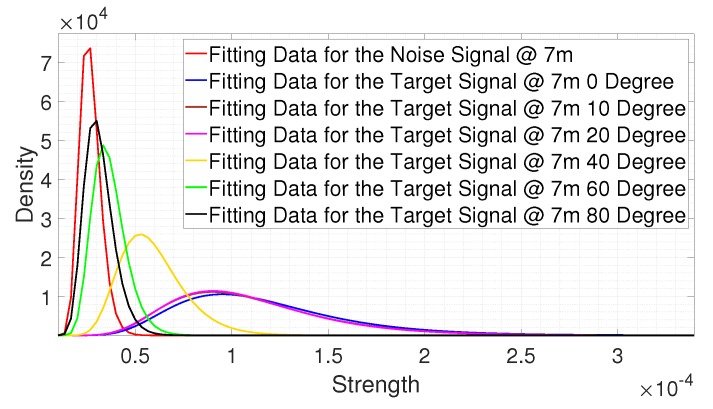
The fitting results for the signals reflected from a person at different angles of 7 m.

Figure 12 shows the fitting results where the signal reflected from a person was collected from the different angles at 7 m. In Figure 12, we observed that, as the target moved away from the main lobe, the strength distribution of the signal reflected from a person was shifted to the left. This means that, the farther the distance was from the main lobe, the weaker the target signal. In other words, the SNR decreased with an increase of the angle. This results in an increase of the miss-detection rate, which means the detection ability decreased with an increase of the angle. Thus, the detectable angle can be measured based on the estimated miss-detection rate.

If a person moved to an angle where the estimated miss-detection rate was lower than the desired miss-detection rate, we could define that angle as the detectable angle.

**Figure 13:**
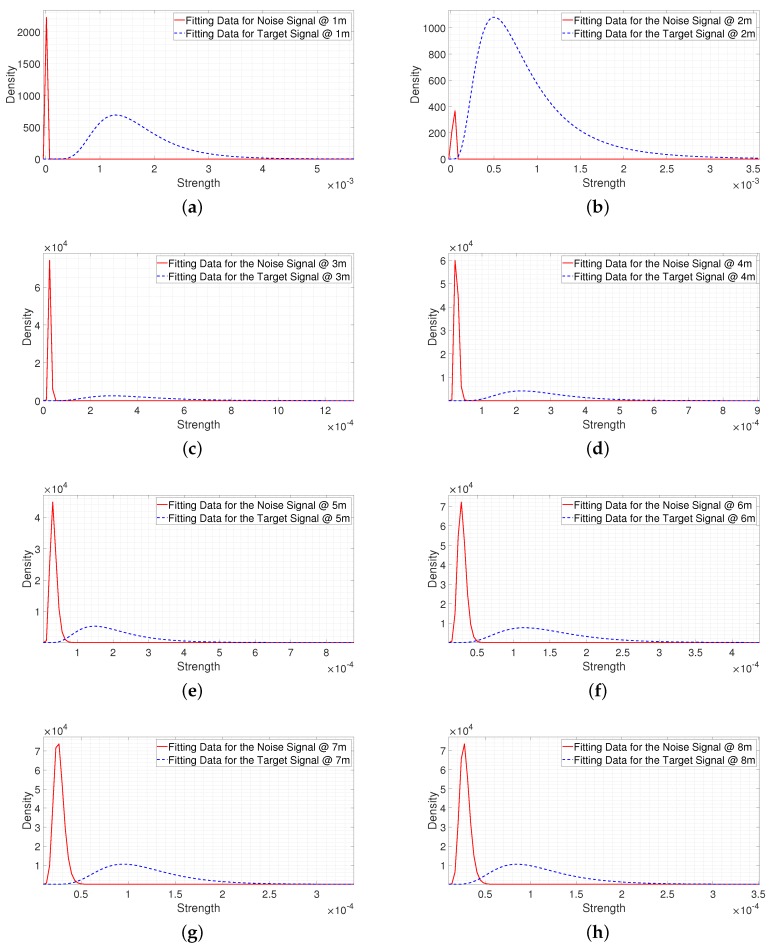
The fitting results from 1 m to 8 m in a light clutter environment. (**a**) The fitting result at 1 m. (**b**) The fitting result at 2 m. (**c**) The fitting result at 3 m. (**d**) The fitting result at 4 m. (**e**) The fitting result at 5 m. (**f**) The fitting result at 6 m. (**g**) The fitting result at 7 m. (**h**) The fitting result at 8 m.

Figure 13 shows the fitting results for the noise signal and the signal reflected from a person in a light clutter environment at the same collection distance from 1 m in 1 m increments. In Figure 13a–d, we observe that, there are no overlapping parts between two fitting results, which means that the signal reflected from a person was much stronger than the noise signal in the range of 1 m to 4 m (a near-region). However, as shown in Figure 13e–h, the overlapping parts increased with the distance, as the signal reflected from a person decreased with the distance. This means the probability that the signal reflected from a person was weaker than the noise signal, which was relatively high, in the range of 5 m to 8 m (a far-region). If these conditions can be considered in determining a threshold, the analysis and prediction results of the cost from a miss-detection or false alarm will be made visible. Also, a threshold can be set according to the focusing point by considering the estimated false alarm and miss-detection rates.

#### 4.1.2. Experiment in a Heavy Clutter Environment

**Figure 14:**
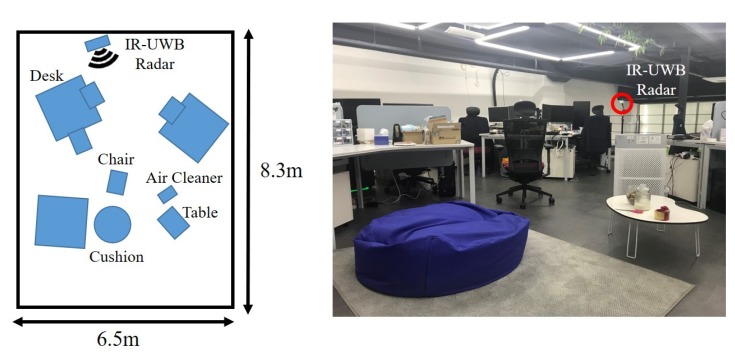
Heavy clutter environment.

Figure 14 shows the experiment environment with heavy clutter. This is an office environment with three big desks, several chairs, and computers. We collected both the noise and target signals from 1 m to 8 m in 1 m increments and analyzed the statistical distribution.

**Figure 15:**
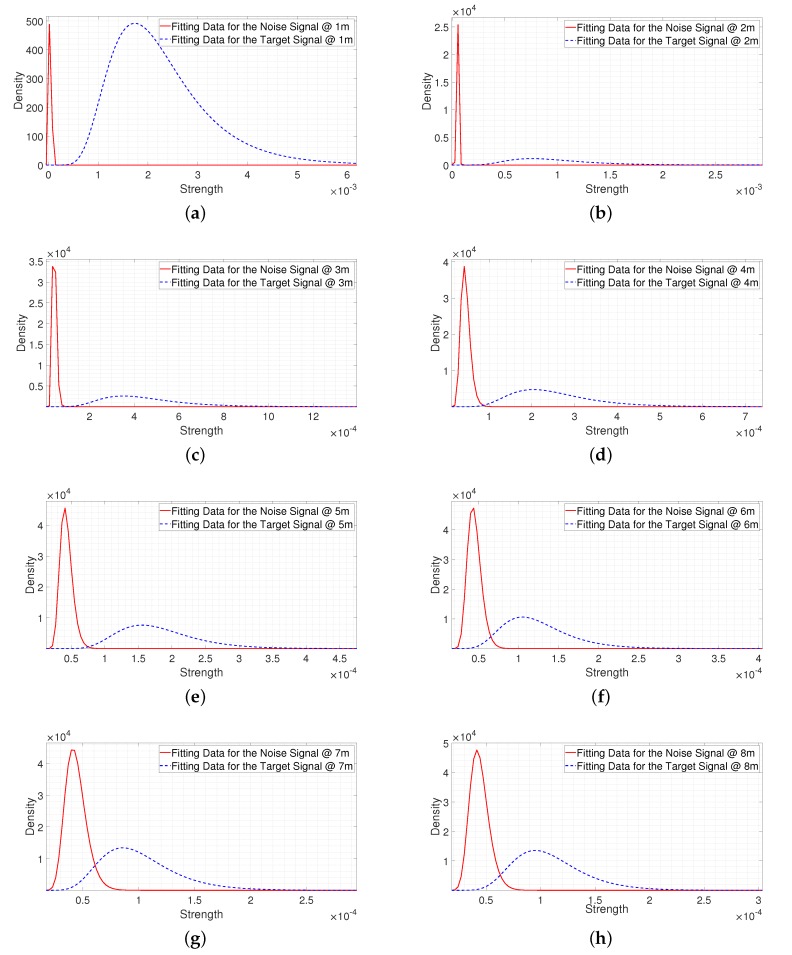
The fitting results from 1 m to 8 m in a heavy clutter environment. (**a**) The fitting result at 1 m. (**b**) The fitting result at 2 m. (**c**) The fitting result at 3 m. (**d**) The fitting result at 4 m. (**e**) The fitting result at 5 m. (**f**) The fitting result at 6 m. (**g**) The fitting result at 7 m. (**h**) The fitting result at 8 m.

Figure 15 shows the fitting results for the noise signal and the signal reflected from a person in a heavy clutter environment at the same collection distance from 1 m in 1 m increments. In Figure 15, we observe that the gap between two fitting results was small compared to the light clutter environment, as heavy clutter results in a low SNR. This means the probability that the signal reflected from a person was weaker than the noise signal was relatively high compared to the light clutter environment. In particular, in the case of 6 m to 8 m, there were many overlapping parts. In this environment, if a threshold is designed only based on the noise signal, where a threshold is designed to reduce the false alarm rate, the probability of miss-detection could be relatively high due to the low SNR. However, if both the noise signal and the signal reflected from a person are considered, a threshold can be designed by considering the estimated miss-detection and false alarm rates. Therefore, a threshold can be designed effectively according to the purpose.

#### 4.1.3. Experiments in Multiple People Environments

**Figure 16:**
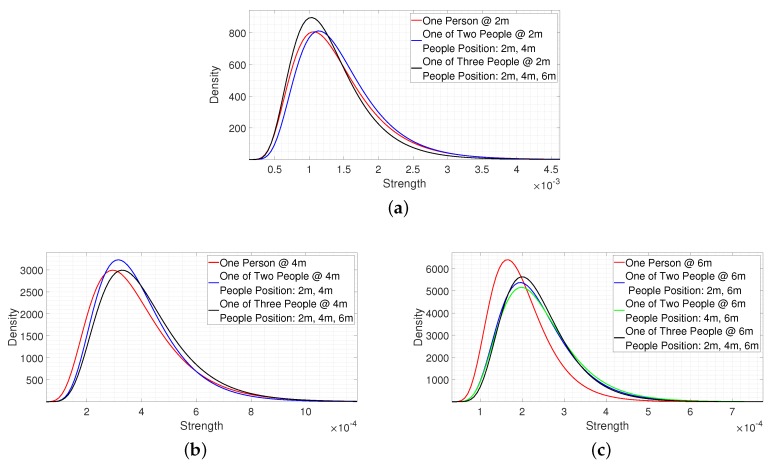
The fitting results in multiple people environments. (**a**) The fitting result at 2 m. (**b**) The fitting result at 4 m. (**c**) The fitting result at 6 m.

Figure 16 shows the fitting results when there are different numbers of people in the detection range. Figure 16a–c are the fitting results for the signal reflected from a person at the same collection distance at 2 m, 4 m, and 6 m.

In Figure 16a, the red line refers to one person at 2 m, the blue line refers to one of two people at 2 m (people were located at 2 m and 4 m), and the black line refers to one of three people at 2 m (people were located at 2 m, 4 m, and 6 m). From Figure 16a, we observe that the statistical distribution of the received signal at 2 m was similar to the one person case even with multiple numbers of people, which means the signal strength of the person was similar. There were no objects that could affect the target signal other than the clutter. The same clutter environment resulted in this. Thus, the target can be detected at this certain distance even with multiple people, as a threshold was designed by considering the signal strength of single person environment, which was similar for the multiple people case.

In Figure 16b, the red line refers to one person at 4 m, the blue line refers to one of two people at 4 m (people were located at 2 m and 4 m), and the black line refers to one of three people at 4 m (people were located at 2 m, 4 m, and 6 m). In Figure 16c, the red line refers to one person at 6 m, the blue line refers to one of two people at 6 m (people were located at 2 m and 6 m), the green line refers to one of two people at 6 m (people were located at 4 m and 6 m), and the black line refers to one of three people at 6 m (people were located at 2 m, 4 m, and 6 m).

From Figure 16b,c, we observe that the statistical distribution of the target signal shifted to the right when there were multiple people in the detection range, which means the signal strength was increased compared to the single person environment. As the received signal could be affected by the multi-paths that were reflected from the people in front, this indicates that the received target signal would be stronger than the threshold, which was designed based on the signal strength of a single person. Thus, the targets can be detected at certain distances. The above results show that the proposed method would work well in multiple people environments.

### 4.2. Performance Analysis

We analyzed the performance of the proposed algorithm through an error rate analysis. The error can be analyzed in two aspects: based on a false alarm rate and based on a miss-detection rate. A false alarm occurs when the noise signal is stronger than the threshold, which can be expressed as follows:

(14) $$\begin{array}{cc} P_{Fa}\left(T_{F}\right)= & P(C>T_{F})\\ = & 1-\int_{0}^{T_{F}}\frac{1}{\sqrt{2\pi }x\sigma_{C}}e^{-\frac{{}^{\left(\ln^{x}-\mu_{C}\right){}^{2}}}{2{\left(\sigma_{C}\right)}^{2}}}dx\\ = & \frac{1}{2}-\frac{1}{2}\mathrm{erf}\left(\frac{\ln^{T_{F}}-\mu_{C}}{\sqrt{2}\sigma_{C}}\right). \end{array}$$

A miss-detection occurs when the human signal is weaker than the threshold. This can be expressed as follows:

(15) $$\begin{array}{cc} P_{Md}\left(T_{F}\right)= & P(H<T_{F})\\ = & \int_{0}^{T_{F}}\frac{1}{\sqrt{2\pi }x\sigma_{H}}e^{-\frac{{}^{\left(\ln^{x}-\mu_{H}\right){}^{2}}}{2{\left(\sigma_{H}\right)}^{2}}}dx\\ = & \frac{1}{2}+\frac{1}{2}\mathrm{erf}\left(\frac{\ln^{T_{F}}-\mu_{H}}{\sqrt{2}\sigma_{H}}\right). \end{array}$$

**Figure 17:**
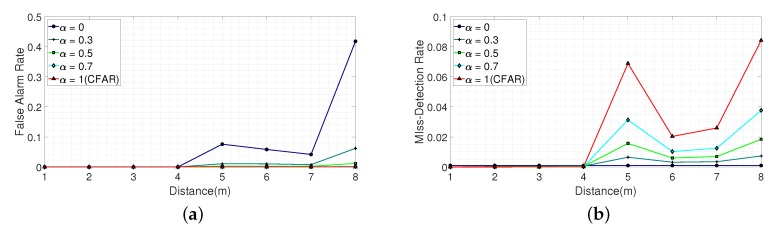
The false alarm rate and miss-detection rate in a light clutter environment according to α. (**a**) False alarm rate according to α. (**b**) Miss-detection rate according to α.

Based on the experimental data, we analyzed the false alarm and miss-detection rates for two types of cluttered environments according to five different values of α, namely, 0, 0.3, 0.5, 0.7, and 1. In the case of α equal to 0, the final threshold $T_{F}$ was set based only on $T_{Md}$, that is, $T_{F}$ was determined by only considering the miss-detection rate. On the other hand, if α was set to 1, the final threshold $T_{F}$ was set based only on $T_{Fa}$, that is, $T_{F}$ is determined by only considering the false alarm rate. In this case, $T_{F}$ was the same with the CFAR algorithm. In the experiments, the target false alarm rate and the target miss-detection rate were set to $10^{-4}$ and $10^{-3}$, respectively. Figure 17 and Table 1, Table 2, Table 3, Table 4 and Table 5 show the estimated false alarm and miss-detection rates for a light clutter environment according to α. We observe that, in the near-region, both the false alarm and miss-detection rates can be further reduced compared to the target values; however, in the far-region they were different due to the low SNR.

Thus, the false alarm was sacrificed in the far-region, when α was equal to 0, that is, the threshold was set to be optimized for the miss-detection. On the other hand, the miss-detection was sacrificed in the far-region when α was equal to 1, that is, the threshold was set to be optimized for the false alarm. Setting α to a value other than 0 and 1 resulted in a lower false alarm rate and miss detection rate in the far-region than when α was 0 and 1, respectively. This is because both the false alarm and miss detection rates were taken into account when determining a threshold, allowing trade-offs between false alarms and miss detection.

**Figure 18:**
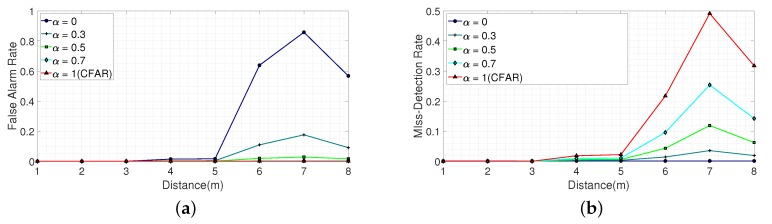
False alarm rate and miss-detection rate in a heavy clutter environment according to α. (**a**) False alarm rate according to α. (**b**) Miss-detection rate according to α.

Figure 18 and Table 6, Table 7, Table 8, Table 9 and Table 10 show the estimated false alarm and miss-detection rates for a heavy clutter environment according to α. This shows that the effect on the false alarm and miss-detection rates caused by the designed threshold according to α was same as the light clutter environment. However, the high SNR region became shorter compared to the light clutter environment, because the heavy clutter decreased the SNR. We observe that, from 1 m to 3 m, both the false alarm and miss-detection rates can be further reduced compared to the target values. However, from 4 m to 8 m, the false alarm was sacrificed when α was equal to 0, and the miss-detection was sacrificed when α was equal to 1, because a threshold was set to be optimized for one condition. We also observe that, from 4 m to 8 m, when α was set to a value other than 0 and 1, the false alarm and miss-detection rates were lower than when α was 0 and 1, respectively. This indicates that trade-offs between false alarms and missed-detections are available, which means the proposed algorithm works in a heavy clutter environment.

The above results indicate that it was possible to set a flexible threshold to suit their purpose by adjusting α. In addition, both the false alarm and miss-detection rates for the determined threshold can be estimated.

### 4.3. Discussion

The validity of the proposed algorithm was verified through the experiments conducted in different environments with different clutter levels and different numbers of people, using an IR-UWB radar. The results demonstrated the effectiveness of the proposed algorithm with respect to the distance of the target, the amount of clutter, and the number of people. In terms of distance, we divided the data into a high SNR region and low SNR region, as the SNR decreased with distance. For the high SNR region, both the false alarm and miss-detection rates can be further reduced compared to the target values. For the low SNR region, a threshold can be determined to allow trade-offs between the false alarm and miss-detection rates. In terms of the amount of clutter, we observed that the SNR decreased as the amount of clutter increased. Thus, in a heavy clutter environment, consideration only on the noise signals in determining a threshold could lead to a relatively high probability of miss-detection due to the low SNR. As the proposed algorithm considered both noise and target signals, it allowed a threshold to be determined according to the intended purpose. In terms of the number of people, we can observe that when there were multiple people, the received signal was affected by the multi-paths that were reflected between people. It results in the strength of the target signal became stronger compared to the single target case, allowing detection in multiple human environments. Therefore, the proposed algorithm can effectively work in multiple people environments. We have also experimentally shown that the proposed algorithm can numerically estimate both the false alarm and miss-detection rates.

## 5. Conclusions

In this paper, we proposed a new algorithm for determining a threshold for IR-UWB radar-based detection applications by taking both the false alarm and miss-detection rates into consideration. As the proposed algorithm considered the target signal as well as the noise signal, it was relatively advantageous in determining a threshold compared to the CFAR algorithm. The proposed algorithm allowed a threshold to be determined according to the focusing point by adjusting the weight factor. Both the miss-detection and the false alarm rates can be estimated for a determined threshold, which makes it possible to analyze the costs from the false alarm and miss-detection rates. In order to verify the validity of the proposed algorithm, we conducted experiments with respect to the distance of the target, the amount of the clutter, and the number of people. The results demonstrated that the proposed algorithm was effective in the tested environments. As one of the applications, the proposed algorithm can be used for measuring the detectable boundary. 

## Figures and Tables

**Table 1:** Performance analysis in a light clutter environment when $P_{Fa}$ = $10^{-4}$, $P_{Md}$ = $10^{-3}$, α = 0.

Distance	1 m	2 m	3 m	4 m
False Alarm Rate	0	$1.1\times 10^{-14}$	$2.3\times 10^{-9}$	$5.3\times 10^{-7}$
Miss-Detection Rate	$1.0\times 10^{-3}$	$1.0\times 10^{-3}$	$1.0\times 10^{-3}$	$1.0\times 10^{-3}$
**Distance**	**5 m**	**6 m**	**7 m**	**8 m**
False Alarm Rate	0.0758	0.0583	0.0417	0.4179
Miss-Detection Rate	$1.0\times 10^{-3}$	$1.0\times 10^{-3}$	$1.0\times 10^{-3}$	$1.0\times 10^{-3}$

**Table 2:** Performance analysis in a light clutter environment when $P_{Fa}$ = $10^{-4}$, $P_{Md}$ = $10^{-3}$, α = 0.3.

Distance	1 m	2 m	3 m	4 m
False Alarm Rate	0	$7.8\times 10^{-12}$	$5.4\times 10^{-8}$	$2.6\times 10^{-6}$
Miss-Detection Rate	$6.3\times 10^{-5}$	$3.6\times 10^{-4}$	$4.4\times 10^{-4}$	$5.7\times 10^{-4}$
**Distance**	**5 m**	**6 m**	**7 m**	**8 m**
False Alarm Rate	0.0108	0.01003	0.0079	0.062
Miss-Detection Rate	0.0065	0.0032	0.0035	0.0073

**Table 3:** Performance analysis in a light clutter environment when $P_{Fa}$ = $10^{-4}$, $P_{Md}$ = $10^{-3}$, α = 0.5.

Distance	1 m	2 m	3 m	4 m
False Alarm Rate	0	$7.6\times 10^{-10}$	$4.6\times 10^{-7}$	$7.3\times 10^{-6}$
Miss-Detection Rate	$3.5\times 10^{-6}$	$1.5\times 10^{-4}$	$2.3\times 10^{-4}$	$3.7\times 10^{-4}$
**Distance**	**5 m**	**6 m**	**7 m**	**8 m**
False Alarm Rate	0.0028	0.0029	0.0024	0.0118
Miss-Detection Rate	0.0157	0.006	0.0069	0.0184

**Table 4:** Performance analysis in a light clutter environment when $P_{Fa}$ = $10^{-4}$, $P_{Md}$ = $10^{-3}$, α = 0.7.

Distance	1 m	2 m	3 m	4 m
False Alarm Rate	0	$8.4\times 10^{-8}$	$4.0\times 10^{-6}$	$2.1\times 10^{-5}$
Miss-Detection Rate	$3.5\times 10^{-8}$	$5.2\times 10^{-5}$	$1.1\times 10^{-4}$	$2.4\times 10^{-4}$
**Distance**	**5 m**	**6 m**	**7 m**	**8 m**
False Alarm Rate	$7.3\times 10^{-4}$	$7.8\times 10^{-4}$	$6.8\times 10^{-4}$	0.0019
Miss-Detection Rate	0.0313	0.0103	0.0125	0.0377

**Table 5:** Performance analysis in a light clutter environment when $P_{Fa}$ = $10^{-4}$, $P_{Md}$ = $10^{-3}$, α = 1 (CFAR algorithm).

Distance	1 m	2 m	3 m	4 m
False Alarm Rate	$1.0\times 10^{-4}$	$1.0\times 10^{-4}$	$1.0\times 10^{-4}$	$1.0\times 10^{-4}$
Miss-Detection Rate	$4.4\times 10^{-16}$	$6.1\times 10^{-6}$	$2.9\times 10^{-5}$	$1.1\times 10^{-4}$
**Distance**	**5 m**	**6 m**	**7 m**	**8 m**
False Alarm Rate	$1.0\times 10^{-4}$	$1.0\times 10^{-4}$	$1.0\times 10^{-4}$	$1.0\times 10^{-4}$
Miss-Detection Rate	0.0689	0.0203	0.0259	0.0842

**Table 6:** Performance analysis in a heavy clutter environment when $P_{Fa}$ = $10^{-4}$, $P_{Md}$ = $10^{-3}$, α = 0.

Distance	1 m	2 m	3 m	4 m
False Alarm Rate	0	0	$1.7\times 10^{-9}$	0.0143
Miss-Detection Rate	$1.0\times 10^{-3}$	$1.0\times 10^{-3}$	$1.0\times 10^{-3}$	$1.0\times 10^{-3}$
**Distance**	**5 m**	**6 m**	**7 m**	**8 m**
False Alarm Rate	0.0164	0.6388	0.8581	0.5677
Miss-Detection Rate	$1.0\times 10^{-3}$	$1.0\times 10^{-3}$	$1.0\times 10^{-3}$	$1.0\times 10^{-3}$

**Table 7:** Performance analysis in a heavy clutter environment when $P_{Fa}$ = $10^{-4}$, $P_{Md}$ = $10^{-3}$, α = 0.3.

Distance	1 m	2 m	3 m	4 m
False Alarm Rate	0	$1.1\times 10^{-15}$	$1.1\times 10^{-6}$	0.0034
Miss-Detection Rate	$7\times 10^{-5}$	$1.4\times 10^{-4}$	$5.1\times 10^{-4}$	0.003
**Distance**	**5 m**	**6 m**	**7 m**	**8 m**
False Alarm Rate	0.0038	0.1091	0.1759	0.0903
Miss-Detection Rate	0.0032	0.0142	0.0354	0.019

**Table 8:** Performance analysis in a heavy clutter environment when $P_{Fa}$ = $10^{-4}$, $P_{Md}$ = $10^{-3}$, α = 0.5.

Distance	1 m	2 m	3 m	4 m
False Alarm Rate	0	$9.9\times 10^{-13}$	$4.1\times 10^{-6}$	0.0012
Miss-Detection Rate	$4.5\times 10^{-6}$	$2.4\times 10^{-5}$	$3.1\times 10^{-4}$	0.0055
**Distance**	**5 m**	**6 m**	**7 m**	**8 m**
False Alarm Rate	0.0014	0.0192	0.0281	0.0161
Miss-Detection Rate	0.0061	0.0431	0.1189	0.0623

**Table 9:** Performance analysis in a heavy clutter environment when $P_{Fa}$ = $10^{-4}$, $P_{Md}$ = $10^{-3}$, α = 0.7.

Distance	1 m	2 m	3 m	4 m
False Alarm Rate	$1.7\times 10^{-16}$	$1.3\times 10^{-9}$	$1.5\times 10^{-5}$	$4.5\times 10^{-4}$
Miss-Detection Rate	$6.1\times 10^{-8}$	$2.1\times 10^{-6}$	$1.8\times 10^{-4}$	0.0093
**Distance**	**5 m**	**6 m**	**7 m**	**8 m**
False Alarm Rate	$4.9\times 10^{-4}$	0.0026	0.0033	0.0023
Miss-Detection Rate	0.0107	0.0958	0.25395	0.1422

**Table 10:** Performance analysis in a heavy clutter environment when $P_{Fa}$ = $10^{-4}$, $P_{Md}$ = $10^{-3}$, α = 1 (constant false alarm rate algorithm (CFAR) algorithm).

Distance	1 m	2 m	3 m	4 m
False Alarm Rate	$1.0\times 10^{-4}$	$1.0\times 10^{-4}$	$1.0\times 10^{-4}$	$1.0\times 10^{-4}$
Miss-Detection Rate	$8.9\times 10^{-15}$	$6.5\times 10^{-9}$	$7.2\times 10^{-5}$	0.0184
**Distance**	**5 m**	**6 m**	**7 m**	**8 m**
False Alarm Rate	$1.0\times 10^{-4}$	$1.0\times 10^{-4}$	$1.0\times 10^{-4}$	$1.0\times 10^{-4}$
Miss-Detection Rate	0.022	0.2171	0.4916	0.3176
